# Alteration of Trop-2 expression in breast cancer cells by clinically used therapeutic agents and acquired tamoxifen resistance

**DOI:** 10.1007/s12282-022-01389-3

**Published:** 2022-07-27

**Authors:** Jing Zhu, Wenwen Wu, Yukiko Togashi, Naoe Taira Nihira, Yoshikazu Johmura, Dajiang Zhu, Makoto Nakanishi, Yasuo Miyoshi, Tomohiko Ohta

**Affiliations:** 1 Department of Translational Oncology, St. Marianna University Graduate School of Medicine, 2-16-1, Sugao, Miyamae-ku, Kawasaki, 216-8511 Japan; 2 Department of Breast Medicine, Foshan Maternity and Child Healthcare Hospital, Southern Medical University, Foshan, China; 3 Department of Cancer and Senescence Biology, Cancer Research Institute, Kanazawa University, Kanazawa, Japan; 4 Division of Cancer Cell Biology, Institute of Medical Science, University of Tokyo, Tokyo, Japan; 5 Division of Breast and Endocrine Surgery, Department of Surgery, Hyogo College of Medicine, Hyogo, Japan

**Keywords:** Trop-2, Sacituzumab govitecan, Tamoxifen, TFEB

## Abstract

**Background:**

Sacituzumab govitecan is an antibody–drug conjugate that delivers SN-38, an active metabolite of irinotecan, to the target molecule, trophoblast cell-surface antigen 2 (Trop-2). It is a promising drug for triple-negative breast cancer and is anticipated to be effective for luminal breast cancer. The efficacy of the agent relies on the expression of Trop-2 rather than its intracellular function. However, conditions that alter the Trop-2 expression have not been well investigated.

**Methods:**

We tested a range of clinically related treatments for their effect on Trop-2 expression in cultured breast cancer cell lines.

**Results:**

The expression level of Trop-2 differed among cell lines, independent of their subtypes, and was highly variable on treatment with kinase inhibitors, tamoxifen, irradiation, and chemotherapeutic agents including irinotecan. While inhibitors of AKT, RSK, and p38 MAPK suppressed the Trop-2 expression, tamoxifen treatment significantly increased Trop-2 expression in luminal cancer cell lines. Notably, luminal cancer cells with acquired resistance to tamoxifen also exhibited higher levels of Trop-2. We identified transcription factor EB (TFEB) as a possible mechanism underlying tamoxifen-induced elevation of Trop-2 expression. Tamoxifen triggers dephosphorylation of TFEB, an active form of TFEB, and the effect of tamoxifen on Trop-2 was prevented by depletion of TFEB. A luciferase reporter assay showed that Trop-2 induction by TFEB was dependent on a tandem E-box motif within the Trop-2 promoter region.

**Conclusions:**

Overall, these results suggest that the effectiveness of sacituzumab govitecan could be altered by concomitant treatment and that tamoxifen could be a favorable agent for combined therapy.

**Supplementary Information:**

The online version contains supplementary material available at 10.1007/s12282-022-01389-3.

## Introduction

Triple-negative breast cancer (TNBC) is the most aggressive subtype of breast cancer and is characterized as estrogen and progesterone receptor-negative and human epidermal growth factor receptor 2 (HER2) negative. Patients with advanced or metastatic TNBC exhibit poor survival outcomes. However, recent advances in breast cancer treatments, including immune-oncology (IO) agents, poly (ADP-ribose) polymerase inhibitors, and antibody–drug conjugates (ADCs), such as sacituzumab govitecan, may dramatically improve the situation.

Sacituzumab govitecan delivers SN-38, an active metabolite of the topoisomerase I inhibitor irinotecan, to cancer cells expressing the target molecule trophoblast cell-surface antigen 2 (Trop-2). The drug has shown remarkable activity in TNBC, with an objective response rate (ORR) of approximately 30–35% [1–3]. The recent phase 3 ASCENT trial, a global open-label randomized trial, compared sacituzumab govitecan with chemotherapy in 468 heavily pretreated, metastatic TNBC patients. The trial demonstrated promising results with a median progression-free survival of 5.6 months, compared to 1.7 months in the control, with a hazard ratio (HR) of 0.41. The median overall survival was 12.1 months compared to 6.7 months (HR 0.48) [3]. The ORR was 35% for sacituzumab govitecan and 5% for standard chemotherapy. Thus, sacituzumab govitecan is expected to be a game-changer for the treatment of advanced TNBC.

In addition to TNBC, sacituzumab govitecan has shown encouraging results when treating hormone receptor-positive/HER2-negative metastatic luminal breast cancer that had progressed after endocrine-based therapy and at least one prior chemotherapy; achieving an ORR of 31.5% [4]. Based on these results, a randomized phase III trial is currently ongoing (NCT03901339 and TROPiCS-02). Because Trop-2 is expressed in a broad range of cancers, the efficacy of sacituzumab govitecan in cancers other than breast cancer has been evaluated in various clinical studies [5, 6].

Trop-2, also known as tumor-associated calcium signal transducer 2 (TACSTD2), is a transmembrane glycoprotein with high homology to the epithelial cell adhesion molecule (EpCAM), also known as Trop-1/TACSTD1 [7–9]. Trop-2 undergoes intramembrane proteolysis and is cleaved into a large extracellular fragment and a short intracellular fragment [10]. The intracellular fragment binds to β-catenin and contributes to the transcriptional activation of cyclin D1 and c-Myc, which accelerates proliferation and promotes epithelial hyperplasia and stem cell self-renewal [10]. Trop-2 increases intracellular calcium concentration, decreases fibronectin binding and cell adhesion, and increases cell motility [11, 12]. In addition to breast cancer, Trop-2 is overexpressed in many types of epithelial cancers, including colorectal, oral, nasopharyngeal, pancreatic, gastric, gallbladder, lung, ovarian, endometrial, and cervical cancers. A meta-analysis of these cancers concluded that Trop-2 overexpression was associated with poor overall survival [13].

Unlike previous ADCs, such as trastuzumab emtansine (T-DM1), the antibody–drug conjugation of sacituzumab govitecan is mediated by a pH-sensitive hydrolyzable linker. The cleavable linker increases the bystander effect by releasing the free payload SN-38 to kill the surrounding Trop-2 negative cells, even in tumors that heterogeneously express Trop-2 [14]. Theoretically, the efficacy of the agent simply relies on the expression level of Trop-2 rather than its intracellular function. Therefore, the estimation of Trop-2 expression in individual clinical settings is critical. However, conditions that alter the expression level of Trop-2 have not been well investigated, although its downstream functions have been relatively well characterized. In the present study, we investigated the alteration in Trop-2 expression under multiple conditions that are used in clinical breast cancer therapies. The results demonstrate that Trop-2 expression is highly variable, which can affect the efficacy of sacituzumab govitecan.

## Materials and methods

### Cell lines and culture conditions

Breast cancer cell lines MCF-7, T47D, ZR-75-1, BT474, HCC1500, HCC1937, MDA-MB-361, MDA-MB-231, MCF10A normal human mammary epithelial cells, HEK293T human embryonic kidney cells, and HeLa human cervical adenocarcinoma cells were obtained from ATCC along with authentication, and cultured according to the supplier’s instructions. Cell lines maintained for more than 20 passages were further authenticated using short tandem repeat genotyping. All cells were routinely monitored for mycoplasma infection using a Mycoplasma Detection Set (TaKaRa, Japan). Cells stably expressing BRCA1-specific short hairpin RNA (shRNA) in doxycycline (Dox)-inducible manner were established by lentiviral infection with CS-RfA-ETBsd comprising targeting sequences, followed by selection with blasticidin, as previously described [15]. The cells were treated with 1 µg/ml Dox for 48 h prior to experimentation. Tamoxifen-resistant cell lines were established by culturing the cells in a medium containing IC_90_ doses of 4-hydroxytamoxifen (4-OHT) for 24 h, washed, and further cultured in a medium containing IC_50_ doses of 4-OHT for approximately one month for MCF-7 and 3 months for T47D and ZR-75-1 cells, until cells recovered to exponential growth states. Cells were then maintained with 5 µM 4-OHT unless otherwise described. Cells treated with ionizing irradiation (IR) were exposed to the indicated doses of X-ray irradiation and cultured for the stated times before analysis. The chemical agents that were used are listed in Table 1. The concentration of the agents was determined by preliminary examinations based on the manufacturer's instructions and on their effects on appropriate markers.

**Table 1:** Chemical agents used in the study

Name	Company	Concentration
Beta-estradiol	Sigma-Aldrich (St.Louis MO)	10 nM
Heregulinβ-1	PeproTech (NJ)	10 ng/ml
Epidermal growth factor	Sigma-Aldrich (St.Louis MO)	100 ng/ml
Forskolin	Sigma-Aldrich (St.Louis MO)	10 uM
Lapatinib	Selleckchem (Houston TX)	1 uM
Everolimus	Selleckchem (Houston TX)	100 nM
MK2206	Cayman (Ann Arbor MI)	10 uM
BI-D1870	Abcam (Cambridge UK)	10 uM
SB202190	Selleckchem (Houston TX)	10 uM
4-Hydroxytamoxifen	Sigma-Aldrich (St.Louis MO)	100 nM ~ 10 uM
Epirubicin	Tronto Research Chem (Toronto CAN)	200 nM
Docetaxel hydrate	Sanofi S.A. (Paris FRA)	50 nM ~ 5 uM
Cisplatin	Combi-Blocks (San Diego CA)	10 ug/ml
Olaparib	JS Research Chemicals Trading (Wedel DEU)	10 uM
Irinotecan (CPT-11)	Sigma-Aldrich (St.Louis MO)	1.5 ~ 4.5 uM

### siRNAs and transfection

siRNA oligonucleotides targeting Trop-2 (#1: s8364 and #2: s8366), TFEB (#1: s224819 and #2: s15496), and a non-targeting control (4390847) were purchased from Thermo Fisher Scientific, and BRCA1 (D-003461-08), and a non-targeting control (D-001210-05) were purchased from Dharmacon. RNA duplexes at a final concentration of 10 nM were transfected into cells using Lipofectamine RNAiMAX (Invitrogen, CA, USA) and analyzed 48 h after transfection.

### Immunoblotting

Cell lysates were prepared with 0.5% NP-40 buffer (50 mM Tris–HCl [pH 7.5], 0.5% Nonidet P-40, 150 mM NaCl, 50 mM NaF, 1 mM dithiothreitol, 1 mM NaVO_3_, 1 mM PMSF, 2 µg/ml aprotinin, 2 µg/ml leupeptin, 10 µg/ml trypsin inhibitor, and 150 µg/ml benzamidine) and subjected to immunoblotting, as previously described [15, 16]. Benzonase nuclease (125 U/ml; Millipore, MA, USA) and 2 mM MgCl_2_ were supplemented for detection of nuclear proteins. The relative protein expression levels of Trop-2 were measured using a densitometer and normalized to tubulin. The following antibodies were used: rabbit monoclonal antibody against Trop-2 (Abcam, Cambridge, UK), AKT (Cell Signaling Tech, MA, USA), SQSTM1/p62 (D5E2; Cell Signaling Tech), rabbit polyclonal antibody against ERα (Millipore), BRCA1 (C20; Santa Cruz, CA, USA), HER2 (Cell Signaling Tech), pAKT (phospho-Ser473 AKT; Cell Signaling Tech), TFEB (Cell Signaling Tech), pRSK (Pan phospho-Ser221, Ser227, Ser218, Ser232; Invitrogen), LC3 (2775; Cell Signaling Tech), mouse monoclonal antibody against γH2AX (JBW301, Millipore), RSK1 (A-10; Santa Cruz), RSK2 (E-1; Santa Cruz), β-actin (6276; Abcam), and α-tubulin (DM1A; Richard-Allan Scientific, MI, USA).

### RT-qPCR

Total RNA from each sample was isolated using an RNeasy kit (QIAGEN, GmbH Hilden, Germany) according to the manufacturer's instructions. Relative levels of Trop-2 mRNA and β-actin mRNA were measured using One Step TB Green^®^ (TaKaRa) and analyzed on the StepOnePlus™ Real-Time PCR System (Applied Biosystems, CA, USA) using the following primers: Trop-2: forward primer, 5ʹ-GGACATCAAGGGCGAGTCTCTA-3ˊ; reverse primer, 5ʹ-AGGCGCTTCATGGAGAACTTCG-3ʹ; β-actin mRNA: forward primer, 5ʹ-GACCTCTATGCCAACACAGT-3ʹ; reverse primer, 5ʹ-AGTACTTGCGCTCAGGAGGA-3ʹ. Each reaction was performed in triplicate, and the data were analyzed using the comparative Ct method and normalized using the β-actin expression in each sample.

### Luciferase assay

The human *Trop-2 (TACSTD2)* promoter region − 838 to − 1 bp (*Luc1*), − 724 to − 1 bp (*Luc2*), and − 543 to − 1 bp (*Luc3*) upstream of the transcription start site were amplified and subcloned into the pGL4.10-luciferase reporter vector (E6651; Promega,). The E-box mutant was generated by site-directed mutagenesis and the sequence was verified by Sanger sequencing. The pcDNA3.1-TFEB plasmid was purchased from Addgene. pcDNA3-GSK3β was a generous gift from Dr. Yue Xiong, University of North Carolina, Chapel Hill. HEK293T cells were co-transfected with the luciferase reporter plasmid, pCMV-LacZ (631719; TaKaRa), and either empty pcDNA3.1 vector or pcDNA3.1-TFEB with or without pcDNA3-GSK3β, using the standard calcium phosphate precipitation method. After 48 h, luciferase activities were measured using the Luciferase Reporter Assay System (E1500; Promega, WI, USA), according to the manufacturer’s instructions and normalized to the β-galactosidase activity determined by the β-galactosidase enzyme assay system (E2000; Promega).

### Cell viability assay

Appropriate number of cells (1 × 10^5^ for MCF7 and 8 × 10^4^ for T47D and ZR-75-1) were seeded into 96-well plate. After 24 h, cells were exposed to indicated doses of 4-OHT and further cultured for 6 or 7 days. Cells were then collected, and viability was assessed in triplicate using Cell Titer-Blue® (Promega) according to the manufacturer’s instructions.

### Statistical analyses

Statistical analyses were performed using a two-tailed Student’s *t*-test or one-way ANOVA using GraphPad Prism 8 Software. *P* < 0.05 was considered to be significant. Data were expressed as mean ± standard deviation.

## Results

### Trop-2 expression varies among cell lines

**Fig. 1:**
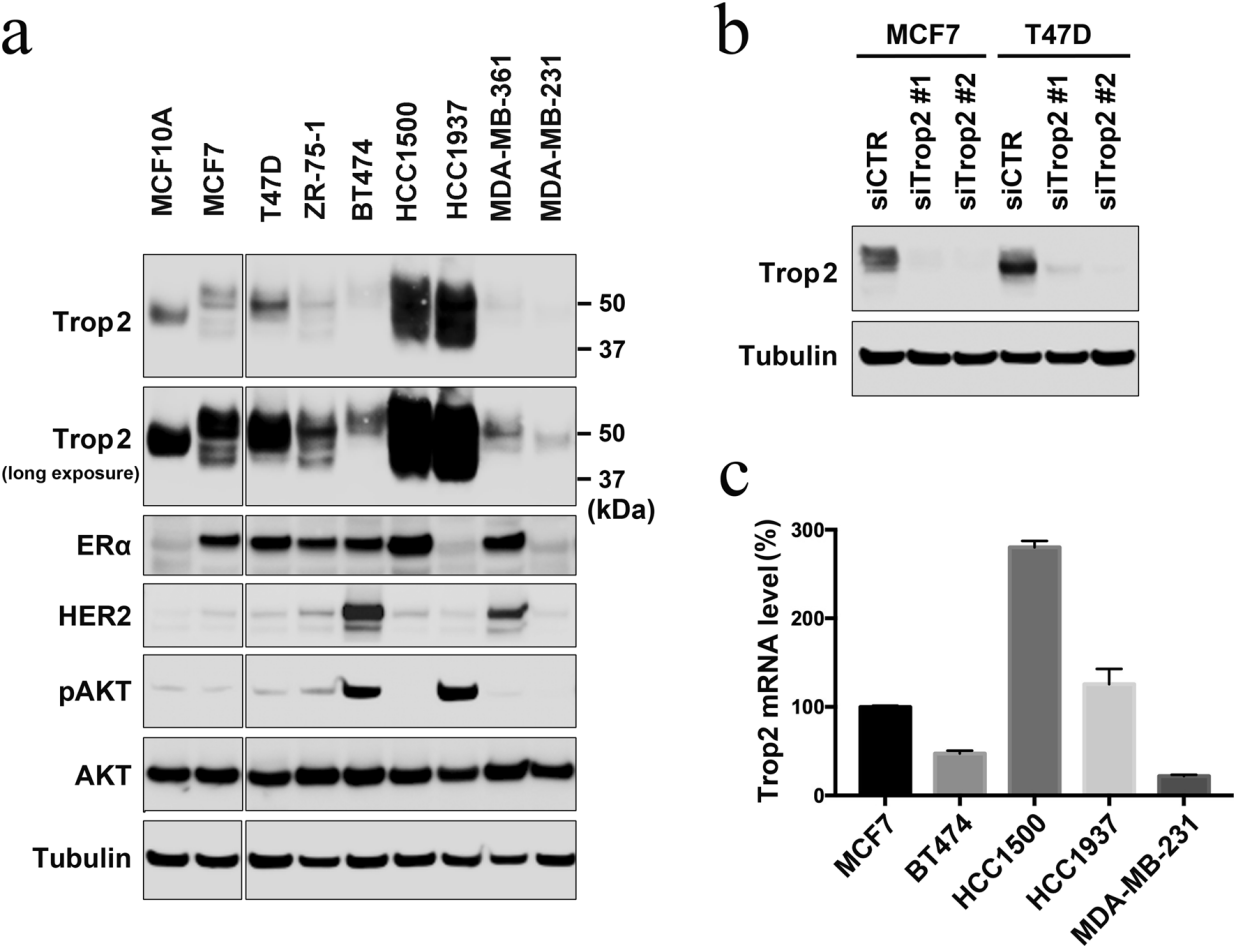
Trop-2 expression in cell lines. **a** Exponentially growing normal breast epithelial MCF-10A cells and the indicated breast cancer cells were subjected to immunoblotting with the indicated antibodies. Tubulin was included as a loading control. **b** MCF-7 and T47D cells were transfected with control siRNA (siCTR) or two different siRNA specific to Trop-2 (siTrop2), and subjected to immunoblotting with anti-Trop-2 antibody. **c** The indicated breast cancer cells in their exponentially growing state were subjected to RT-qPCR. Relative Trop-2 mRNA levels from triplicate experiments are shown with standard deviation (S.D)

We first analyzed the protein expression level of Trop-2 in various breast cancer cell lines and normal breast epithelial cell line MCF-10A by immunoblotting (Fig. 1a). The expression levels of estrogen receptor α (ERα), HER2, and AKT phosphorylated at Ser473 (pAKT) were also analyzed to verify their characteristics. While luminal HCC1500 and triple-negative HCC1937 cells expressed the highest levels of Trop-2, the expression level was the lowest in the two luminal-HER2 type breast cancer cell lines, BT474, and MDA-MB-361, as well as in triple-negative MDA-MB-231 cells. MCF10A and luminal type cell lines MCF-7, T47D and ZR-75-1 expressed moderate levels of Trop-2. The specificity of the antibody was confirmed by siRNA knockdown of Trop-2 (Fig. 1b). These results indicate that the expression level of Trop-2 varies among cell lines and is likely to be independent of the subtypes of breast cancer, although we did not evaluate this in HER2-enriched cell lines.

We next investigated the mRNA expression level of Trop-2 in the representative cell lines. The mRNA expression levels correlated well with the protein expression levels in these cell line; HCC1500 and HCC1937 expressed high levels, whereas BT474 and MDA-MB-231 expressed low levels of Trop-2 mRNA (Fig. 1c). The result suggests that the protein expression level of Trop-2 is mainly due to gene expression rather than protein stability. The smear like mobility shift observed in the Trop-2 immunoblot of HCC1500 and HCC1937 cells (Fig. 1a) was possibly due to glycosylation, as previously reported [17]. The high expression levels of Trop-2 in HCC1937 cells may be due to their BRCA1 status; HCC1937 cells are defective in BRCA1. Because it has been speculated that BRCA1 status may affect the efficacy of sacituzumab govitecan [18], we investigated the effect of BRCA1 depletion on Trop-2 expression in other cell lines. However, the depletion of BRCA1 only slightly increased the Trop-2 expression in MCF7 and T47D cells, with Trop-2 expression decreased or unchanged in the other cell lines (Supplementary Fig. S1). This is consistent with the clinical data that sacituzumab govitecan benefits patients with metastatic TNBC, regardless of BRCA1 status [18].

### Effects of clinically used therapeutic agents on Trop-2 expression

Because the expression level of Trop-2 varied significantly among the cell lines, we considered whether the agents used in clinical therapies could affect the expression of Trop-2, and consequently affect the efficacy of sacituzumab govitecan. To explore the effect of clinically relevant conditions, we treated breast cancer cells with estrogen, growth factors, chemotherapeutic agents, IR, kinase inhibitors, and tamoxifen.

We first tested the effect of estrogen and growth factors on the luminal-type cell lines MCF7, T47D, ZR-75-1, and HCC1500. Cells were treated with estrogen, heregulin, epidermal growth factor, or forskolin which induces protein kinase A activation, followed by immunoblotting (Supplementary Fig. S2a and b). No significant effect on Trop-2 expression was observed.

**Fig. 2:**
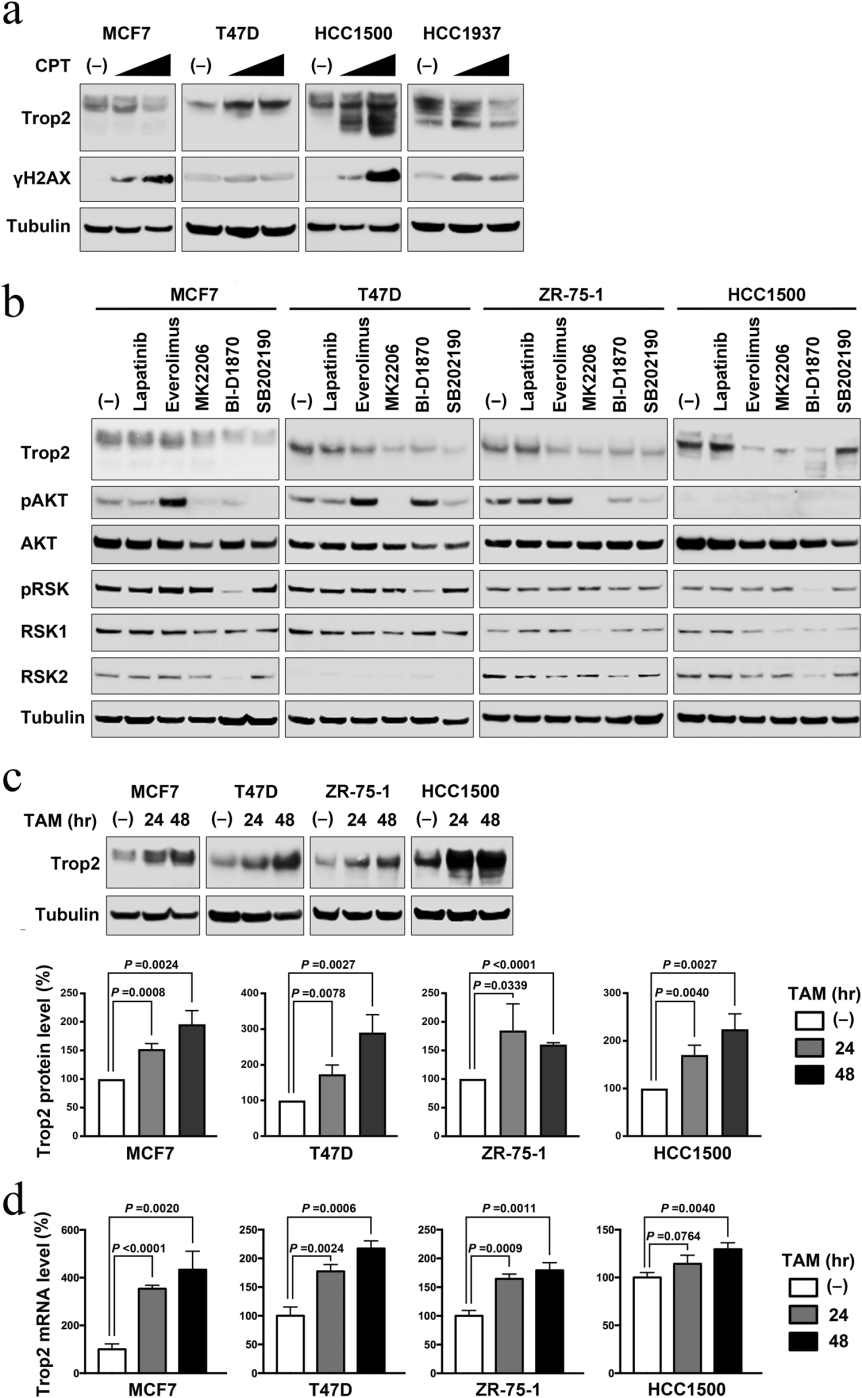
Effects of clinically used agents on Trop-2 expression. **a** Indicated cells were either untreated or treated with 1.5 or 4.5 μM of irinotecan (CPT) for 24 h, and subjected to immunoblotting with the indicated antibodies. γH2AX was examined as a marker of DNA damages. **b** Indicated luminal breast cancer cells were either untreated or treated with 1 μM of lapatinib, 100 nM of everolimus, 10 μM of MK2206, 10 μM of BI-D1870, or 10 μM of SB202190 for 24 h, and subjected to immunoblotting with the indicated antibodies. Phosphorylated AKT and RSK were analyzed as downstream targets. Representative data from two independent experiments are shown (see also Supplementary Fig. S3a). **c** Indicated cells were either untreated or treated with 100 nM (MCF-7 and T47D) or 500 nM (ZR-75-1 and HCC1500) of 4-OHT (TAM) for 24 or 48 h, and subjected to immunoblotting with the indicated antibodies. Representative data (upper panel) and averages ± S.D. of the relative Trop-2 levels normalized with tubulin from three independent experiments (lower panel) are shown. **d** Cells treated as in **c** were subjected to RT-qPCR. Relative Trop-2 mRNA levels from triplicate experiments are shown with S.D. Statistical significances were calculated using Student’s *t*-test

We next examined the effect of chemotherapeutic agents on the luminal cancer cell lines MCF7 and T47D, and the triple-negative cell lines MDA-MB-231 and HCC1937 (Supplementary Fig. S2c). The effects of the agents, including epirubicin, taxane, cisplatin, and olaparib, varied among the cell lines tested. Trop-2 expression was decreased or unaffected by chemotherapeutic agents in most cases. However, treatment with irinotecan markedly increased the expression of Trop-2 in T47D and HCC1500 cells (Fig. 2a). This could be important for the effect of sacituzumab govitecan, whose efficacy relies on irinotecan metabolite SN-38 and Trop-2 expression.

The decrease in Trop-2 observed in MDA-MB-231 and HCC1937 cells after treatment with some chemotherapeutic agents may be triggered by DNA damage. Therefore, we tested whether treatment with IR caused similar results. MCF7, T47D, MDA-MB-231, and HCC1937 cells were irradiated and subsequently examined for their Trop-2 expression level. The Trop-2 expression levels were significantly decreased after 10 Gy IR, especially in MDA-MB-231 and HCC1937 cells (Supplementary Fig. S2d), suggesting that DNA damage may trigger the suppression of Trop-2 expression.

We next tested the effect of kinase inhibitors on Trop-2 expression in the luminal cells MCF7, T47D, ZR-75-1, and HCC1500 (Fig. 2b and Supplementary Fig. S3a). In addition to the generally used tyrosine kinase inhibitor lapatinib and mTORC1 inhibitor everolimus, we included other inhibitors of major kinase pathways, the AKT inhibitor MK2206, RSK inhibitor BI-D1870, and p38 MAPK inhibitor SB202190. MK2206 and BI-D1870 significantly suppressed Trop-2 expression in all four cell lines tested. SB202190 suppressed Trop-2 expression in three cell lines. In contrast, lapatinib did not affect Trop-2 expression in any of the cell lines tested.

Among the therapeutic conditions tested, the most dramatic and possibly clinically important change in Trop-2 expression was observed with tamoxifen treatment. Trop-2 protein expression levels were significantly increased after treatment with tamoxifen for 24 and 48 h in all four luminal cancer cell lines tested (Fig. 2c). The viability of the cells was not affected by this short-term treatment. We evaluated the mRNA expression levels of Trop-2 and found these were also elevated after tamoxifen treatment in all four cell lines and correlated well with the protein expression levels (Fig. 2d), suggesting that the upregulation was due to gene expression rather than protein stability.

### Trop-2 expression is upregulated in tamoxifen-resistant luminal cancer cell lines

**Fig. 3:**
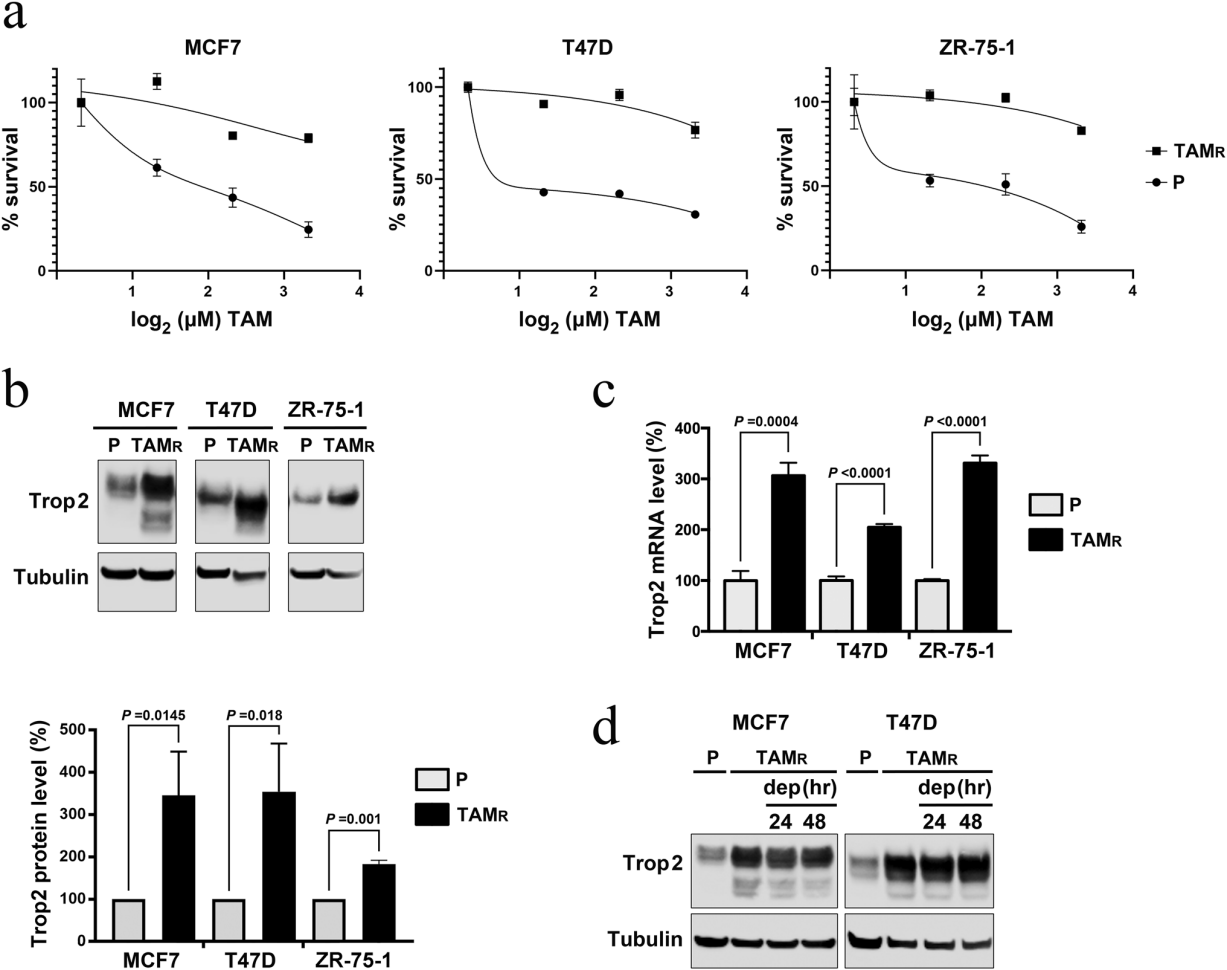
Trop-2 expression is upregulated in tamoxifen-resistant luminal cancer cells. **a** Cells resistant to tamoxifen (TAM_R_, solid square) or tamoxifen-sensitive parental cells (P, solid circle) were cultured for 6 days (MCF-7 and T47D) or 7 days (ZR-75-1) with indicated doses (0–10 μM) of 4-OHT (TAM), and the cell viabilities were measured with Cell Titer Blue reagent. Averages ± S.D. of absorbance were derived from triplicate experiments. **b** Total lysates from the indicated exponentially growing cells were subjected to Trop-2 immunoblotting. Representative data (upper panel) and averages ± S.D. of the relative Trop-2 levels normalized with tubulin from three independent experiments (lower panel) are shown. **c** Cells as in **b** were subjected to RT-qPCR. Relative Trop-2 mRNA levels from triplicate experiments are shown with S.D. **d** Tamoxifen-resistant MCF-7 and T47D cells constantly cultured with 4-OHT were incubated with a medium depleted with 4-OHT for the indicated times and subjected to Trop-2 immunoblotting together with parental cell lysates. Statistical significances were calculated using Student’s* t*-test

In current clinical settings, sacituzumab govitecan is used as a second- or later-line treatment after breast cancer acquires resistance to first-line treatment. Therefore, the tamoxifen-induced elevation of Trop-2 prompted us to investigate whether acquiring tamoxifen resistance also affects Trop-2 expression. Previously, it was reported that tamoxifen-resistant TMX2-28 cells expressed lower levels of Trop-2 mRNA than parental MCF-7 cells [19]. However, TMX2-28 is a clone derived from MCF-7 [20] and its characteristics may depend on its specific genetic background. Therefore, we established three multiclonal luminal cancer cell lines, MCF-7, T47D, and ZR-75-1, which were resistant to tamoxifen (Fig. 3a). In all three cell lines, Trop-2 expression was two or three times higher in tamoxifen-resistant cells than in the parental tamoxifen-sensitive cells, at both protein (Fig. 3b) and mRNA (Fig. 3c) levels. The expression in the tamoxifen-resistant cells remained elevated after withdrawal of tamoxifen showing that this was not a transient effect of tamoxifen (Fig. 3d).

### TFEB mediates tamoxifen-induced Trop-2 expression

**Fig. 4:**
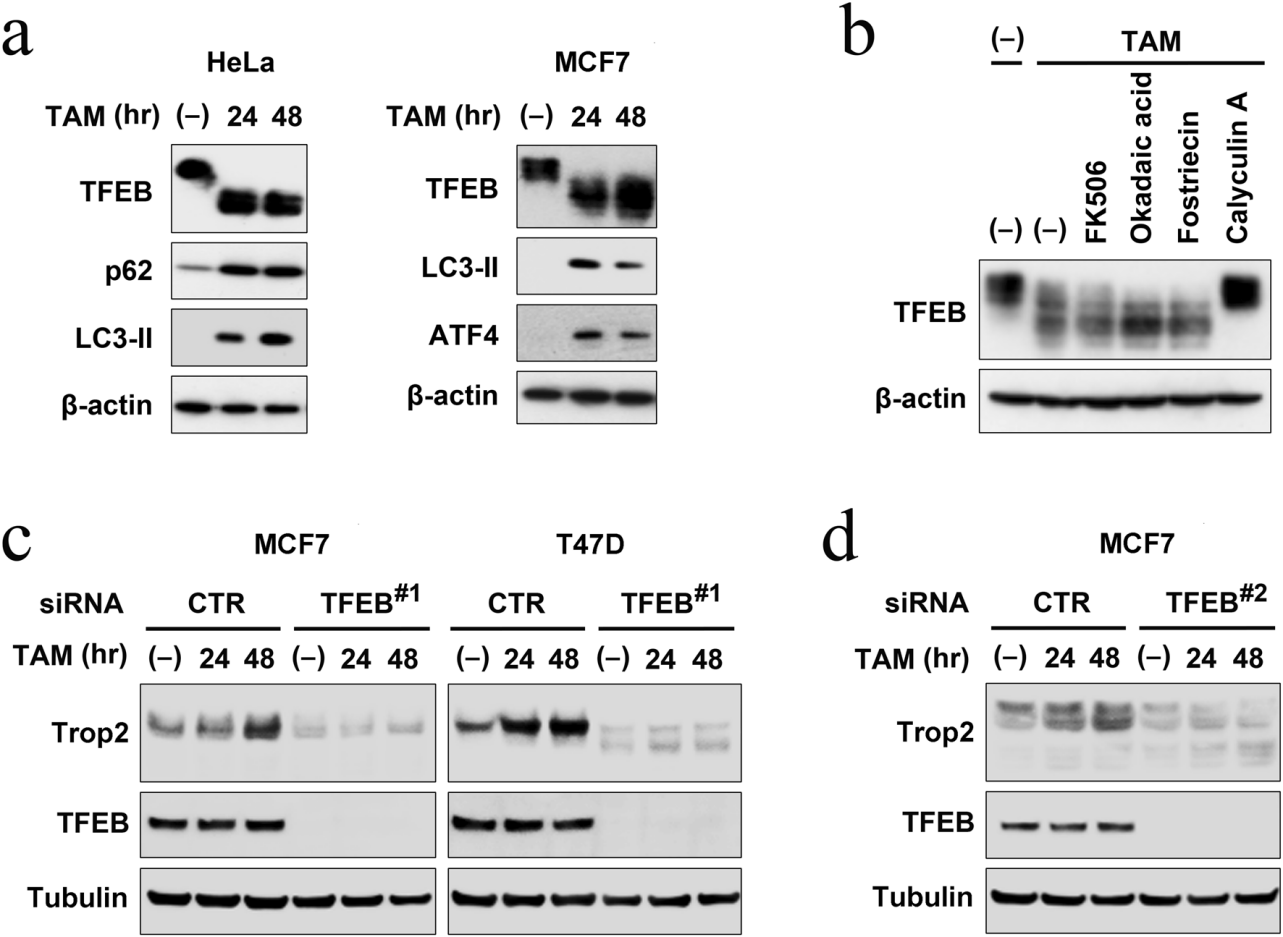
Trop-2 induction by tamoxifen is mediated by TFEB. **a** HeLa or MCF-7 cells were treated with 10 μM 4-OHT for the indicated times and the lysates were subjected to immunoblotting with the indicated antibodies. **b** HeLa cells were treated with 4-OHT (10 μM) with or without FK506 (10 μM), okadaic acid (10 μM), or Fostriecin (5 μM) for 2 h or with Calycurin A (5 μM) for 30 min, and subjected to immunoblotting. **c** MCF-7 and T47D cells transfected with either control siRNA or siRNA targeting TFEB (#1), were treated with or without 100 nM 4-OHT for the indicated times, and subjected to immunoblotting. Representative data from two independent experiments are shown (see also Supplementary Fig. S3b). **d** MCF-7 cells were treated as in (c) except that different siRNA targeting TFEB (#2) was used. Representative data from two independent experiments are shown (see also Supplementary Fig. S3c)

To determine the mechanism underlying tamoxifen-induced Trop-2 expression, we searched the promoter region of Trop-2 and found several E-box sequences. E-box is recognized by certain transcription factors including TFEB, a master transcription factor for autophagy [21]. During previous research on Fbxo22-TFEB functional interaction [22], we noticed that tamoxifen treatment induces mobility shifts of TFEB on the gel due to dephosphorylation, an active form of TFEB, accompanied by the induction of autophagy factors p62, LC3-II, and ATF4 (Fig. 4a). Dephosphorylation was inhibited by the phosphatase inhibitor calyculin A (Fig. 4b). These data prompted us to investigate the role of TFEB in tamoxifen-induced Trop-2 expression. We investigated the effects of TFEB depletion using MCF-7 and T47D cells transfected with either control siRNA or siRNA targeting TFEB and treated with 100 nM tamoxifen (Fig. 4c and supplementary Fig. S3b). Immunoblotting showed Trop-2 was induced by tamoxifen in cells treated with control siRNA. At lower concentrations of tamoxifen, dephosphorylation of TFEB could not be detected by mobility shift on the gel. Remarkably, depletion of TFEB suppressed the expression level of Trop-2 and its upregulation by tamoxifen in both MCF7 and T47-D cells. Similar results were also observed with a different siRNA, targeting an independent sequence in TFEB, arguing against off-target effects (Fig. 4d and supplementary Fig. S3c). Thus, we concluded that tamoxifen-induced Trop-2 expression is mediated by TFEB.

### TFEB promotes Trop-2 transactivation via tandem E-box

**Fig. 5:**
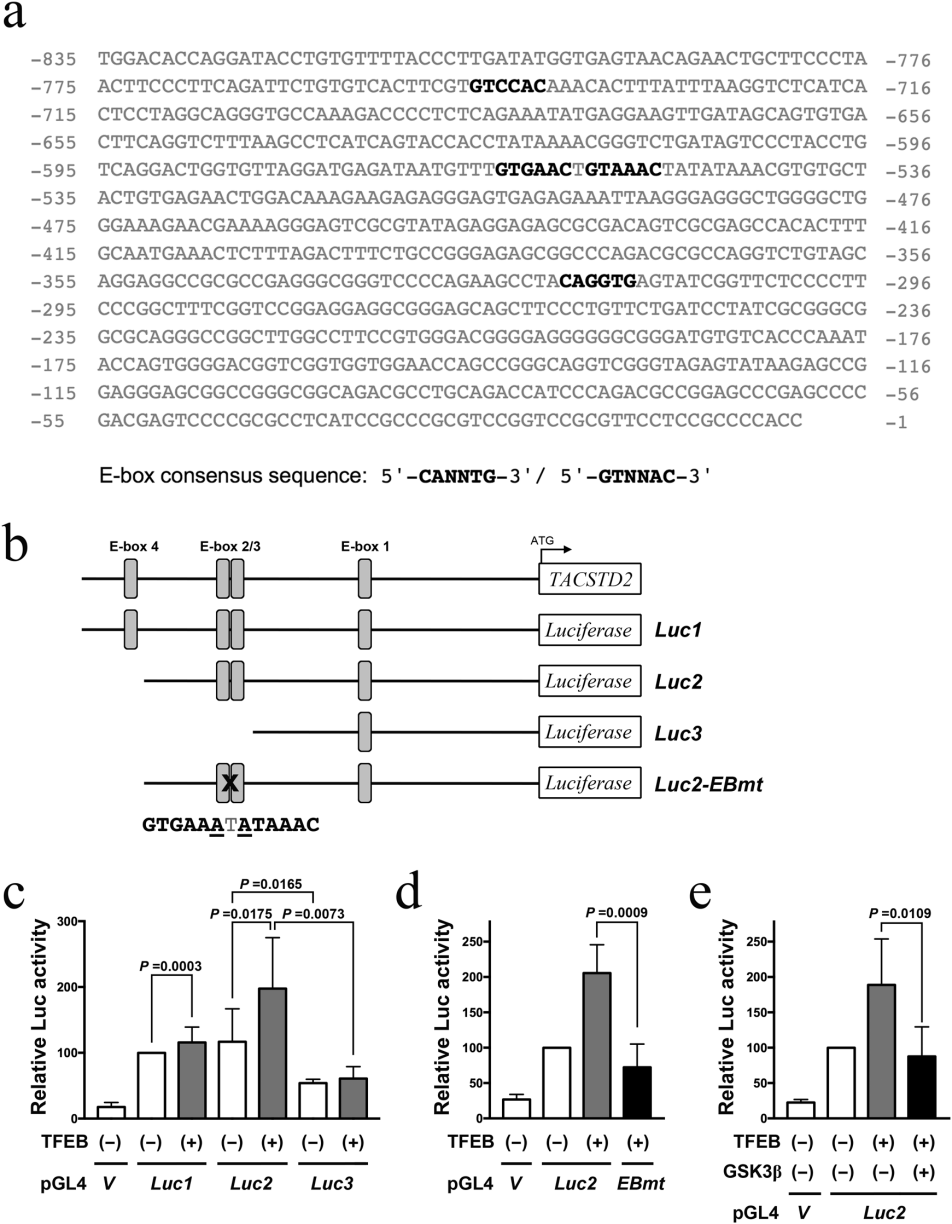
Trop-2 is transactivated by TFEB in a manner dependent on a tandem E-box motif. **a** Sequence of human *TACSTD2* (*Trop-2*) 5′-UTR. E-box consensus sequences are shown in bold. **b** Schematic diagram of the luciferase reporter constructs fused to *TACSTD* promoter used in the study. The mutated residues (underline) in the tandem E-box motif in *Luc2-EBmt* are shown below. **c** HEK293T cells were transfected with control luciferase reporter vector (V) or indicated reporter constructs (*Luc1, 2,* and *3*) with either empty pcDNA3.1 vector (-) or pcDNA3.1-TFEB. After 48 h the cell lysates were subjected to a luciferase assay. **d** Luciferase assay was carried out as in **c** with a reporter mutated in the tandem E-box motif (EBmt) as indicated. **e** Luciferase assay was carried out as in **c** with additional GSK3β expression as indicated. Relative luciferase activities from three independent experiments are shown as averages ± S.D. Statistical significances were calculated using one-way ANOVA

To determine whether TFEB transactivates the Trop-2 promoter, we employed a luciferase reporter assay. The *Trop-2 (TACSTD2)* promoter region upstream of the transcription start site comprises four possible E-box motifs, including one tandem E-box sequence (Fig. 5a). We created three different constructs Luc1, Luc2, and Luc3 comprising E-boxes 1–4, 1–3, or only 1, respectively, fused to the pGL4.10-luciferase reporter (Fig. 5b). These constructs were transfected, with or without TFEB, into HEK 293 T cells. The activity was the highest in Luc2, followed by Luc1, and was significantly reduced in Luc3, indicating that the tandem E-box motif is most critical for Trop-2 expression (Fig. 5c). In addition, TFEB significantly enhanced the activity of Luc1 and Luc2 but not Luc3, indicating that TFEB induces Trop-2 expression via the tandem E-box motif. To confirm this effect, we created a Luc2 construct with mutations in tandem E-box sequences (Luc2-EBmt, Fig. 5b). The mutations significantly suppressed the luciferase activity (Fig. 5d). It has been reported that GSK3β phosphorylates and inhibits TFEB [23–25]; we, therefore, tested whether GSK3β affects TFEB-enhanced Trop-2 induction and found that additional expression of GSK3β prevented the enhancement activity mediated by TFEB expression (Fig. 5e).

## Discussion

In the present work, we showed that the expression level of Trop-2 differed among cell lines and was highly variable under clinically relevant conditions. Although most of the chemotherapeutic agents, IR, and kinase inhibitors suppressed the expression, irinotecan in some cell lines and tamoxifen in all the luminal cancer cell lines tested increased Trop-2 expression. These data suggest some clinical implications.

Biomarker analyses in the ASCENT study, investigating Trop-2 expression levels in archival biopsy or surgical specimens, demonstrated that sacituzumab govitecan is more effective for survival and ORR in metastatic TNBC expressing high or medium levels of Trop-2, compared with standard chemotherapy [18]. However, sacituzumab govitecan could also benefit patients with low Trop-2 expression, although the small number of patients precluded definitive conclusions. For example, ORR in sacituzumab govitecan versus standard chemotherapy-treated patients was 44%, 38%, and 22% versus 1%, 11%, and 6% for high, medium, and low Trop-2 expression, respectively. This suggests that Trop-2 expression in archival specimens may not be the best biomarker for classifying sensitivity. The variability of Trop-2 expression shown in our study suggests that the expression of Trop-2, and, therefore, the sensitivity to sacituzumab govitecan, is likely altered by previous treatments. Our results also suggest that it may be possible to benefit from sacituzumab govitecan, even if the current Trop-2 status is low, by combining treatment with other agents, including tamoxifen. Therefore, alteration of Trop-2 expression by previous treatment should be considered when predicting the effect of sacituzumab govitecan.

Of the treatments tested in this study, only irinotecan and tamoxifen increased Trop-2 expression. The upregulation of Trop-2 by irinotecan in T47D and HCC1500 cells has attracted considerable attention. Because govitecan is an active metabolite of irinotecan, the govitecan released from sacituzumab, which attaches to Trop-2 positive cancer cells, may upregulate Trop-2 expression in neighboring cells, thereby enhancing the bystander effect of sacituzumab govitecan. Further research is required to confirm this observation.

The most significant and clinically important observation in the current study is the upregulation of Trop-2 by tamoxifen because it provides a method to improve the efficacy of sacituzumab govitecan treatment, not only for TNBC but also for luminal breast cancer. Whereas the recent development of therapeutic agents remarkably improved the outcome of luminal breast cancer, a considerable population still become resistant to such treatments. Sacituzumab govitecan is currently used as a second- or later-line treatment after initial hormone therapy for such patients, suggesting that many of the patients had been treated with tamoxifen. The tamoxifen-induced upregulation and the upregulation in tamoxifen-resistant cells shown in this study suggest that sacituzumab govitecan may be most beneficial if used sequentially or concurrently with tamoxifen treatment.

We identified TFEB as an upstream regulator of Trop-2, which is activated by tamoxifen. There have been several studies on the regulation of Trop-2 expression in tumors. Trop-2 is epigenetically downregulated by DNA methylation or loss of heterozygosity in lung adenocarcinoma [26]. In contrast, Trop-2 expression is upregulated in many types of cancer, including breast cancer [27]. As transcription factors that regulate Trop-2, cAMP response element-binding protein (CREB) and zinc finger E-box-binding homeobox 1 (ZEB1) have been reported. CREB binds to the promoter of Trop-2 and upregulates Trop-2, which in turn activates CREB via upregulation of calcium intake, thus constituting a positive feedback loop [28]. As CREB is elevated in tamoxifen-resistant breast cancer cells [29], it has been speculated that Trop-2 is upregulated in tamoxifen-resistant cells [28]. Although we showed that tamoxifen-induced Trop-2 expression is mediated by TFEB, the mechanism upregulating Trop-2 in tamoxifen-resistant cells could be different. Thus, CREB is a possible candidate in this case. In contrast to CREB, ZEB1 transcriptionally represses Trop-2, accompanied by epithelial-mesenchymal transition [30]. Depletion of ZEB1 by siRNA resulted in an increase in Trop-2 and epithelial marker CDH1 expression. Interestingly, ZEB1 is a transcription factor that binds to the E-box [31]. Combined with our results, this suggests that TFEB and ZEB1 may antagonistically regulate Trop-2 transcriptional induction via the E-box.

The mechanisms underlying the inhibition of Trop-2 expression under stressful conditions, including IR and chemotherapy, are currently unknown. However, inhibition induced by kinase inhibitors could occur through the activation of GSK3β, which can phosphorylate and inhibit TFEB [23–25]. Because it was reported that AKT, RSK, and p38 MAPK inhibit GSK3β [32–34] and our data showed that inhibitors of these kinases suppressed Trop-2 expression (Fig. 2b), we tested whether GSK3β affects the luciferase activity mediated by the Trop-2 promoter and TFEB. GSK3β inhibited the enhancement in activity mediated by TFEB expression. This observation is consistent with the prediction that kinase inhibitors suppress Trop-2 expression via GSK3β activation.

In conclusion, these results suggest that the effectiveness of sacituzumab govitecan could be altered by concomitant treatment and that tamoxifen could be a favorable agent for combined therapy. Interestingly, TFEB has been reported to induce programmed death-ligand 1 (PD-L1) [35]. Therefore, tamoxifen treatment may have an adjuvant effect on IO therapy in addition to sacituzumab govitecan. The primary limitation of this study was the lack of clinical data supporting the alteration of Trop-2 expression by therapeutic agents, especially tamoxifen. Because neoadjuvant tamoxifen is not a standard treatment, it was difficult to collect appropriate samples for analysis, which prevented us from obtaining clinical data. Future studies are warranted to clarify whether Trop-2 induction occurs after tamoxifen treatment in clinical settings.

## Supplementary Information

Below is the link to the electronic supplementary material.

Supplementary file1 (PDF 825 kb)
