# Supplementary material for: Alteration of Trop-2 expression in breast cancer cells by clinically used therapeutic agents and acquired tamoxifen resistance
Source: Breast Cancer. 2022 Jul 27;29(6):1076–87. doi: 10.1007/s12282-022-01389-3 (PMC9587948; doi:10.1007/s12282-022-01389-3)
Supplement: Supplementary file 1 — Supplementary file1 (PDF 825 kb) [file 12282_2022_1389_MOESM1_ESM.pdf]

Supplementary Information

Alteration of Trop-2 expression in breast cancer cells by clinically used therapeutic agents and acquired tamoxifen resistance

Supplemental information includes 3 figures.

Jing Zhu[1,2], Wenwen Wu[1], Yukiko Togashi[1], Naoe Taira Nihira[1], Yoshikazu Johmura[3], Dajiang Zhu[2], Makoto Nakanishi[4], Yasuo Miyoshi[5] and Tomohiko Ohta[1,*]

[1]Department of Translational Oncology, St. Marianna University Graduate School of Medicine, Kawasaki, Japan, [2]Department of breast medicine, Foshan Maternity ＆ Child Healthcare Hospital, Southern Medical University, Foshan, China. [3]Department of Cancer and Senescence Biology, Cancer Research Institute, Kanazawa University, Kanazawa, Japan, [4]Division of Cancer Cell Biology, Institute of Medical Science, University of Tokyo, Tokyo, Japan, [5]Division of Breast and Endocrine Surgery, Department of Surgery, Hyogo College of Medicine, Hyogo 663-8501, Japan.

*Corresponding author
Tomohiko Ohta
Department of Translational Oncology,
St. Marianna University Graduate School of Medicine
2-16-1, Sugao, Miyamae-ku, Kawasaki, 216-8511, Japan,
Phone: xx-81-44-977-8111
Fax: xx-81-44-976-5964
E-mail: to@marianna-u.ac.jp
ORCID ID: 0000-0002-9700-7342

SUPPLEMENTAL FIGURES

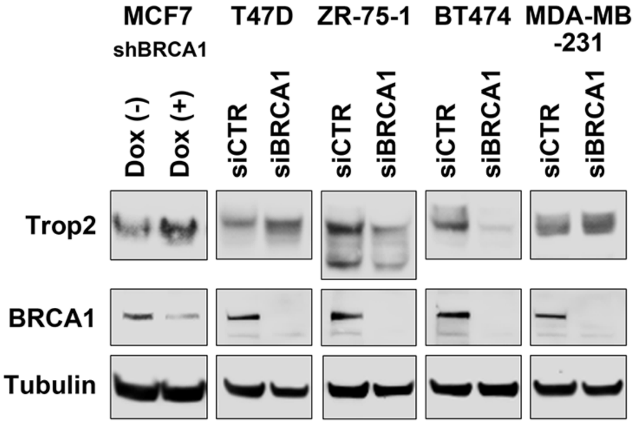

Supplementary Figure S1

The effect of BRCA1 depletion on Trop-2 expression in luminal, luminal/HER2 and triple-negative breast cancer cell lines. MCF-7 cells stably expressing Dox-inducible shRNA to BRCA1 (MCF7-shBRCA1) were treated or untreated with 1 μg/ml Dox for 48 hours. T47D, ZR-75-1, BT474, and MDA-MB-231 cells were transfected either with control siRNA (siCTR) or siRNA specific to BRCA1 (siBRCA1) and incubated for 48 hours. Cells were then subjected to immunoblotting with the indicated antibodies.

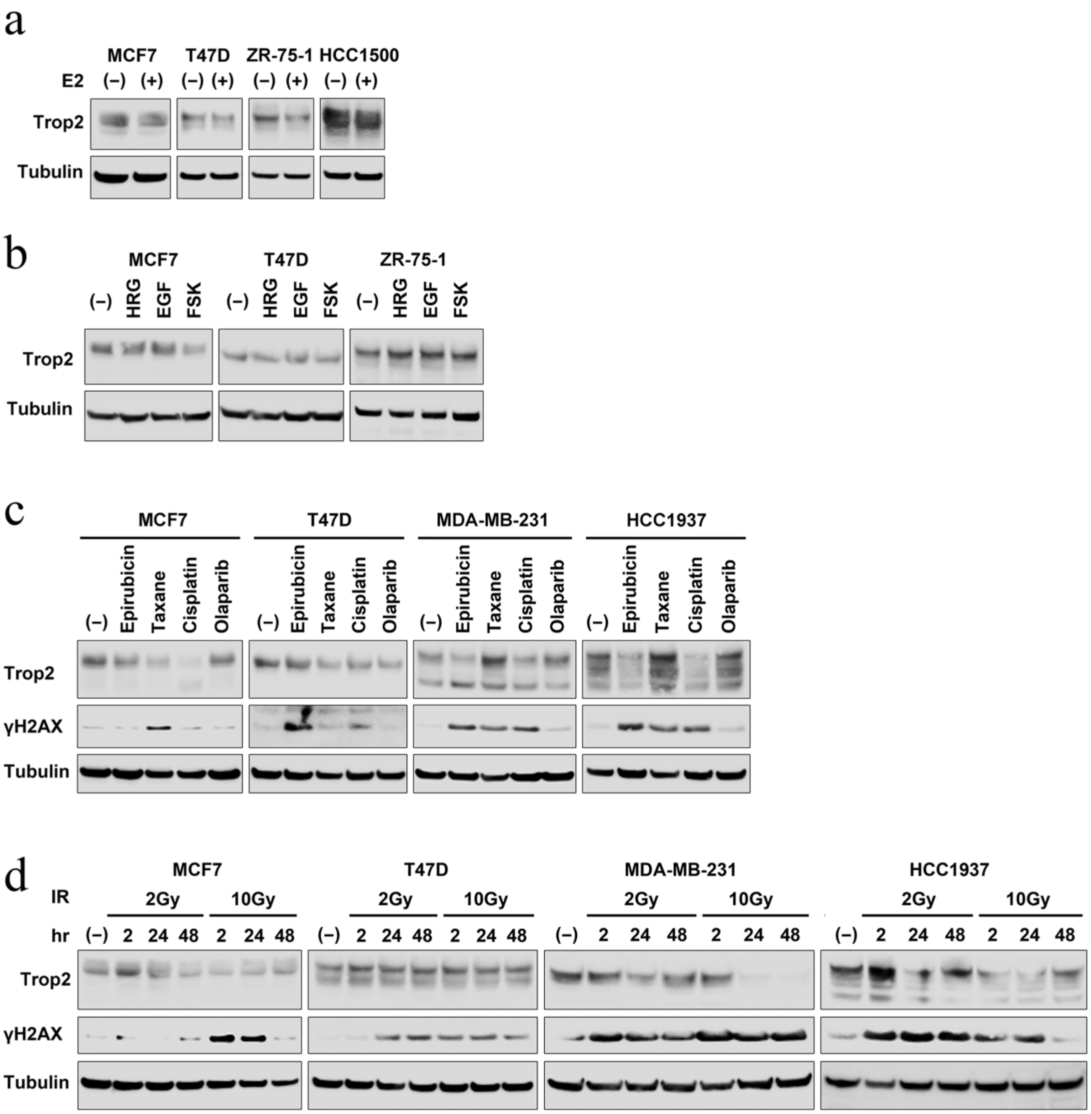

Supplementary Figure S2

The effect of estrogen, growth factors, chemotherapy and IR on Trop-2 expression in breast cancer cell lines. The indicated luminal breast cancer cell lines were either untreated or treated with estrogen (E2) (a), or growth factors heregulin β-1 (HRG), epidermal growth factor (EGF), or forskolin (FSK) (b) for 24 hours and were subjected to immunoblotting with the indicated antibodies. The indicated luminal and triple-negative breast cancer cell lines were either untreated or treated with chemotherapeutic agents epirubicin, taxane, cisplatin, or olaparib for 24 hours (c), or ionizing radiation at the doses shown and incubated for the indicated times (d), and were subjected to immunoblotting. The induction of γH2AX was examined for a marker of DNA damages. The concentration of the agents for the treatment of the cells is shown in Table 1.

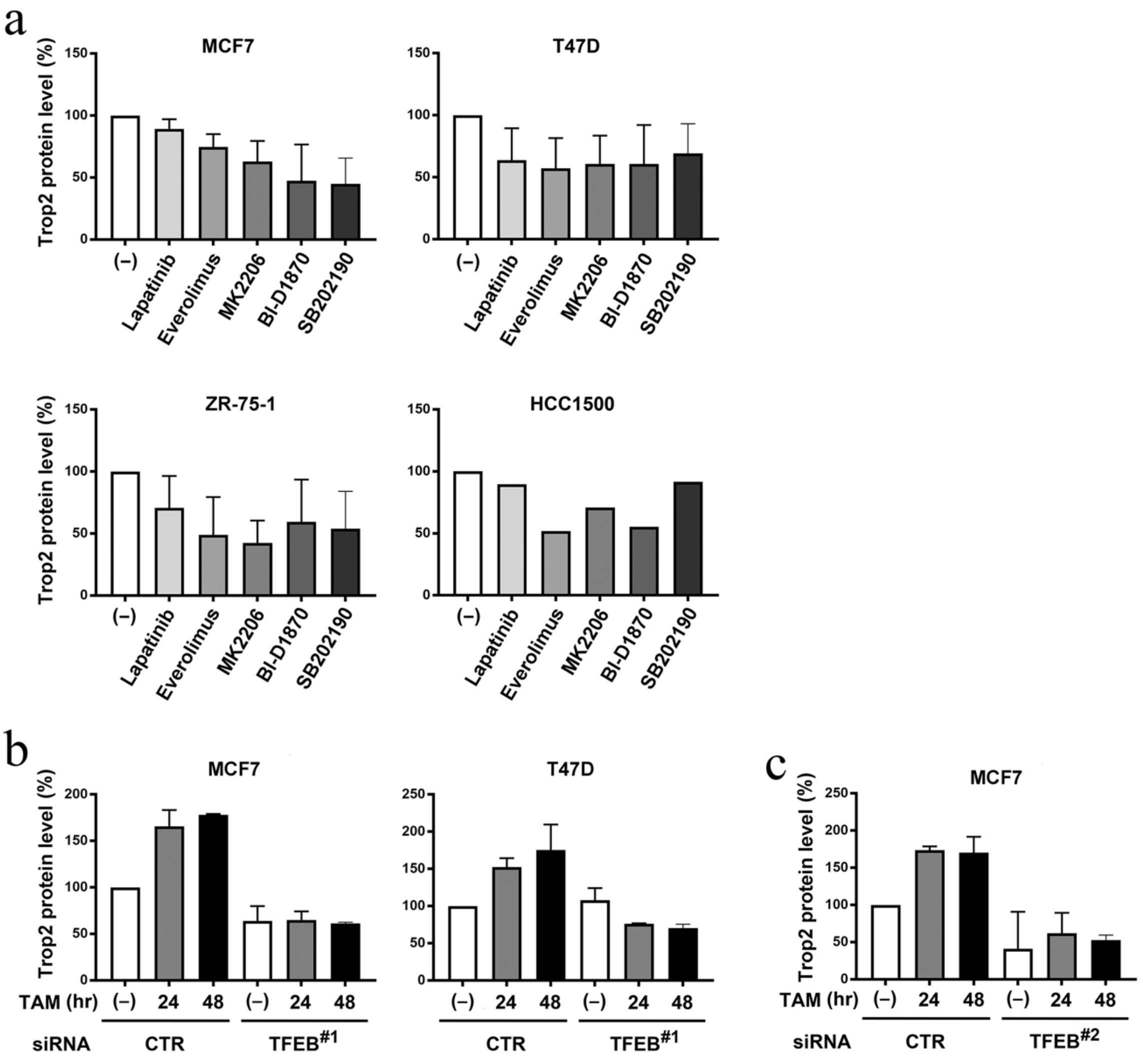

Supplementary Figure S3

Quantification of Trop-2 protein expression. (a) Indicated cells were either untreated or treated with kinase inhibitors and subjected to immunoblotting with Trop-2 antibody as in Figure 2b. Relative Trop-2 protein expression levels normalized to tubulin were shown as averages ± S.D. of two (one for HCC1500) independent experiments. (b and c) Indicated cells were treated and subjected to immunoblotting as in Figure 4c and d. Relative Trop-2 protein expression levels normalized to tubulin were shown as averages ± S.D. of two independent experiments.
